# Supplementary material for: Patchy burn severity explains heterogeneous soil viral and prokaryotic responses to fire in a mixed conifer forest
Source: mSystems. 2025 May 14;10(6):e01749-24. doi: 10.1128/msystems.01749-24 (PMC12172441; doi:10.1128/msystems.01749-24)
Supplement: Supplemental Figures — Figures S1 to S5. [file msystems.01749-24-s0001.pdf]

Supplementary Figures and Captions

Patchy burn severity explains heterogeneous soil viral and prokaryotic responses to fire in a mixed conifer forest

Sara E. Geonczy, Luke S. Hillary, Christian Santos-Medellín,

Jess W. Sorensen, Joanne B. Emerson

mSystems

Supplementary Figure 1. A. DNA yields per gram of soil for each extracted virome at each timepoint, faceted by burn (left) and control (right) plots, with substitution of 0 ng/g soil for all viromes with yields that were below detection limits. Point colors indicate depth of sample. Box boundaries correspond to 25th and 75th percentiles, and whiskers extend to ±1.5x the interquartile range. Horizontal lines indicate whether there was a significant pairwise Wilcox test between timepoints and at which depth (color of lines). Panel background colors highlight pre-burn (green) and post-burn (white) timepoints. **B.** Gravimetric soil moisture percent for each sample at each timepoint, faceted by burn (left) and control (right). Colors, box and whisker parameters, and statistics are the same as in **A**. Timepoints T1-T5 correspond to 14 and 3 days before and 7, 14, and 30 days after the fire, respectively.

Supplementary Figure 2. A. Amount of precipitation in millimeters measured each day between March 01, 2021 to June 30, 2021, with sampling dates highlighted with gray vertical lines and the prescribed burn date highlighted with an orange vertical line. **B-E.** Relationship between pairwise viral community Bray–Curtis similarity and pairwise spatial distance between samples, separated by timepoint to avoid time as a confounding factor [T1 (**B**), T2 (**C**), T3 (**D**), and T4 (**E**)]. Each point is a pair of samples. Trend lines display the least squares linear regression model. Inset statistics correspond to the Pearson's correlation coefficient (r), R^2, the linear regression slope, and the associated P value.

Supplementary Figure 3. A-I. Burn-relevant soil chemical properties for each sample over time, faceted by burn (left) and control (right) plots. Point colors indicate depth of sample (brown 0-3 cm, orange 3-6 cm, same as in **Figure 2**). Box boundaries

correspond to 25th and 75th percentiles, and whiskers extend to ±1.5x the interquartile range. For organic matter, LOI = loss on ignition (method). Panel background colors highlight pre-burn (green) and post-burn (white) timepoints. **J.** Principal component 1 (PC1) of the PCA in panel (**4B**) (the burn severity gradient) with bars representing the loadings along PC1 for each of the burn-relevant soil properties with different colors for either individual or groups of properties. Group colors: yellow = nitrogen properties, green = phosphorus properties, and orange = micronutrients.

Supplementary Figure 4. **A**. Time series temperature measurements from dataloggers placed at each plot (with two sets of data retrieved from plot B1) over the course of the prescribed burn. Figures are faceted by probe depth. Lines are colored by plot location. The red dotted vertical line approximated the beginning of the prescribed burn. **B**. Difference in burn severity between depths (upper depth severity minus lower depth severity) in burned plots, with each bar representing a subplot and timepoint and bars grouped and colored by plot. **C.** Boxplots representing the pairwise Bray-Curtis dissimilarity of viral communities between the two sample depths for each plot replicate at each timepoint. Box boundaries correspond to 25th and 75th percentiles, and whiskers extend to ±1.5x the interquartile range.

Supplementary Figure 5. A/B. Phylum-level relative abundances in 16S rRNA gene profiles of all samples from 0-3 cm (**A**) and 3-6 cm (**B**). Each stacked bar is a sample, and missing bars indicate samples that did not amplify or meet the rarefaction threshold. Stacked bars are faceted by timepoint and grouped within each timepoint by plot. Panel background colors highlight control (gray) and burn (white) plots. The most abundant phyla are colored, with all other low abundance phyla collapsed into “other.” **C-D.**

Hierarchical clustering and heatmap visualizing the correlation analysis of soil properties and relative abundances of prokaryotic phyla (**C**) or vOTU groups with a given phylum-level host prediction (**D**). Gradient of colors indicate Pearson's correlation coefficient. Negative correlations correspond to dark blue and positive correlations correspond to dark red. Asterisks indicate a significant correlation ($p < 0.05$). **E.** Relative abundances of groups of vOTUs according to their phylum-level host predictions for all sequenced viromes. Each stacked bar is a virome. Missing bars indicate viromes that were not sequenced due to low or undetectable DNA yields. Stacked bars are faceted by timepoint and grouped within each timepoint by plot, with the sample depth indicated in the sample label. All other parameters are the same as (**A/B**).

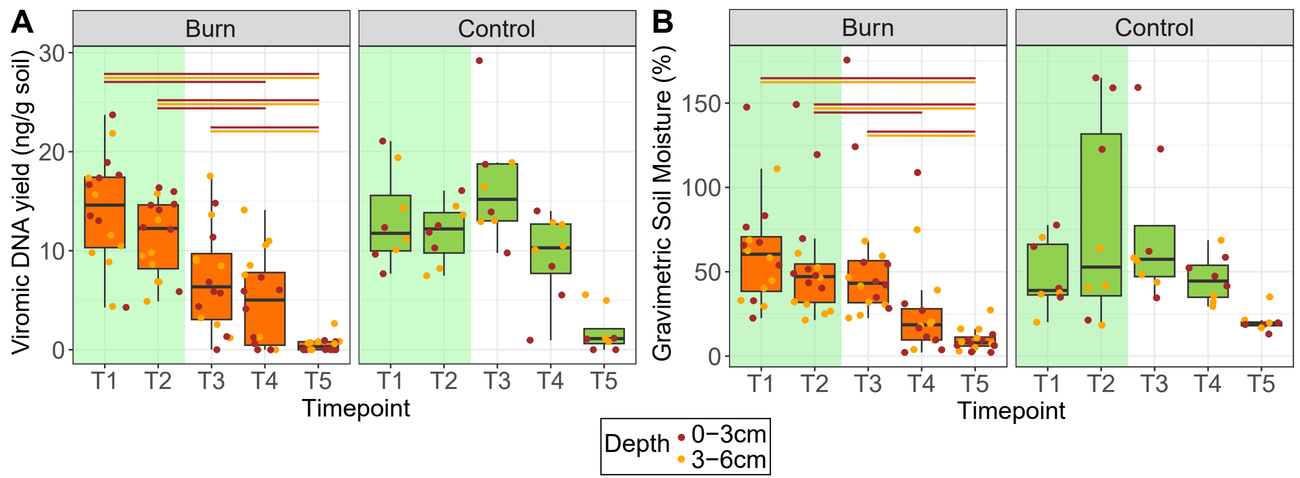
A
Burn
Control
Viromic DNA yield (ng/g soil)
30
20
10
0
T1
T2
T3
T4
T5
Timepoint
B
Burn
Control
Gravimetric Soil Moisture (%)
150
100
50
0
T1
T2
T3
T4
T5
Timepoint
Depth
0−3cm
3−6cm

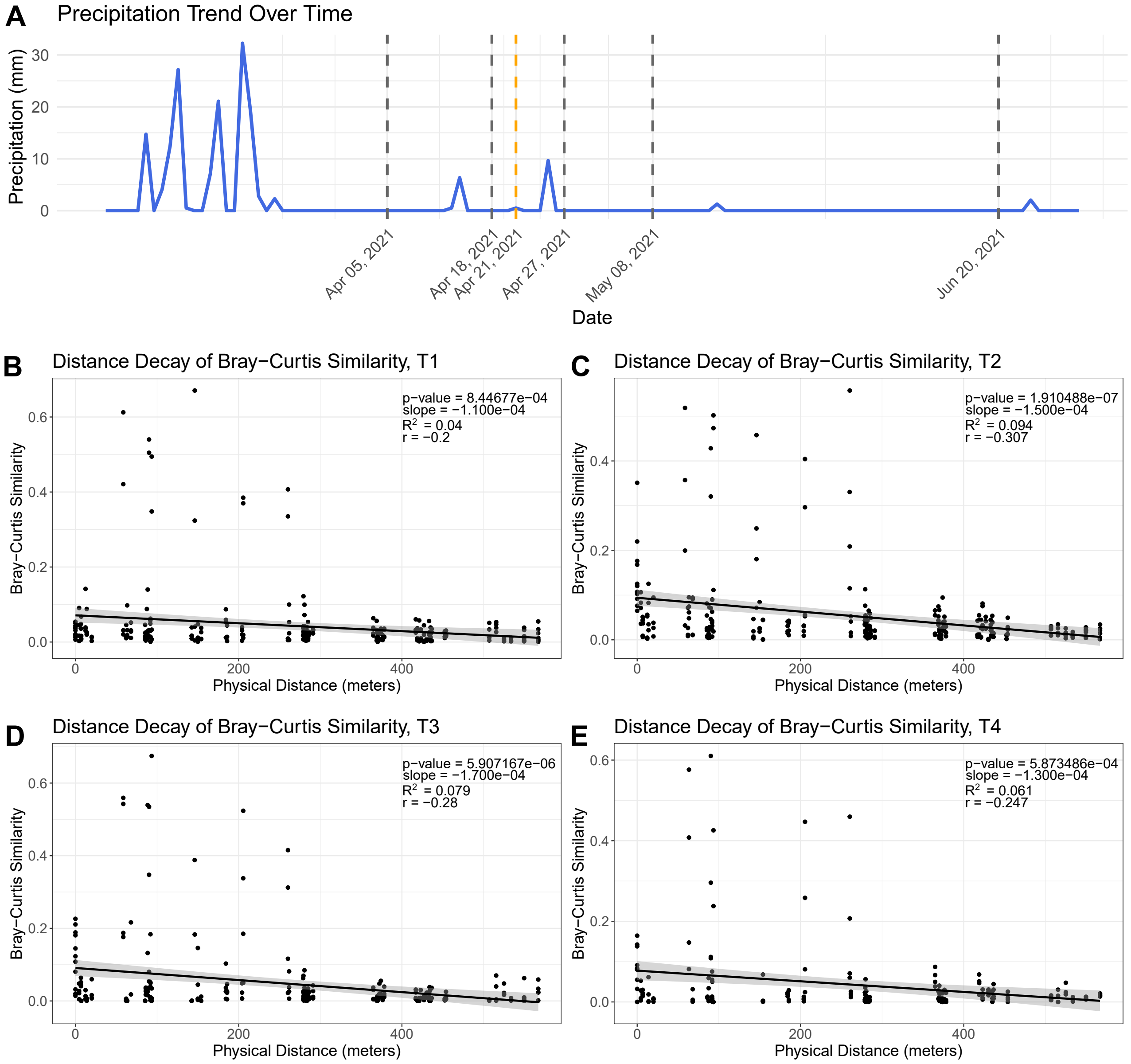
A
Precipitation Trend Over Time
Precipitation (mm)
0
10
20
30
Apr 05, 2021
Apr 18, 2021
Apr 21, 2021
Apr 27, 2021
May 08, 2021
Jun 20, 2021
Date
B
Distance Decay of Bray−Curtis Similarity, T1
p−value = 8.44677e−04
slope = −1.100e−04
R^2 = 0.04
r = −0.2
Bray−Curtis Similarity
0.0
0.2
0.4
0.6
0
200
400
Physical Distance (meters)
C
Distance Decay of Bray−Curtis Similarity, T2
p−value = 1.910488e−07
slope = −1.500e−04
R^2 = 0.094
r = −0.307
Bray−Curtis Similarity
0.0
0.2
0.4
0
200
400
Physical Distance (meters)
D
Distance Decay of Bray−Curtis Similarity, T3
p−value = 5.907167e−06
slope = −1.700e−04
R^2 = 0.079
r = −0.28
Bray−Curtis Similarity
0.0
0.2
0.4
0.6
0
200
400
Physical Distance (meters)
E
Distance Decay of Bray−Curtis Similarity, T4
p−value = 5.873486e−04
slope = −1.300e−04
R^2 = 0.061
r = −0.247
Bray−Curtis Similarity
0.0
0.2
0.4
0.6
0
200
400
Physical Distance (meters)

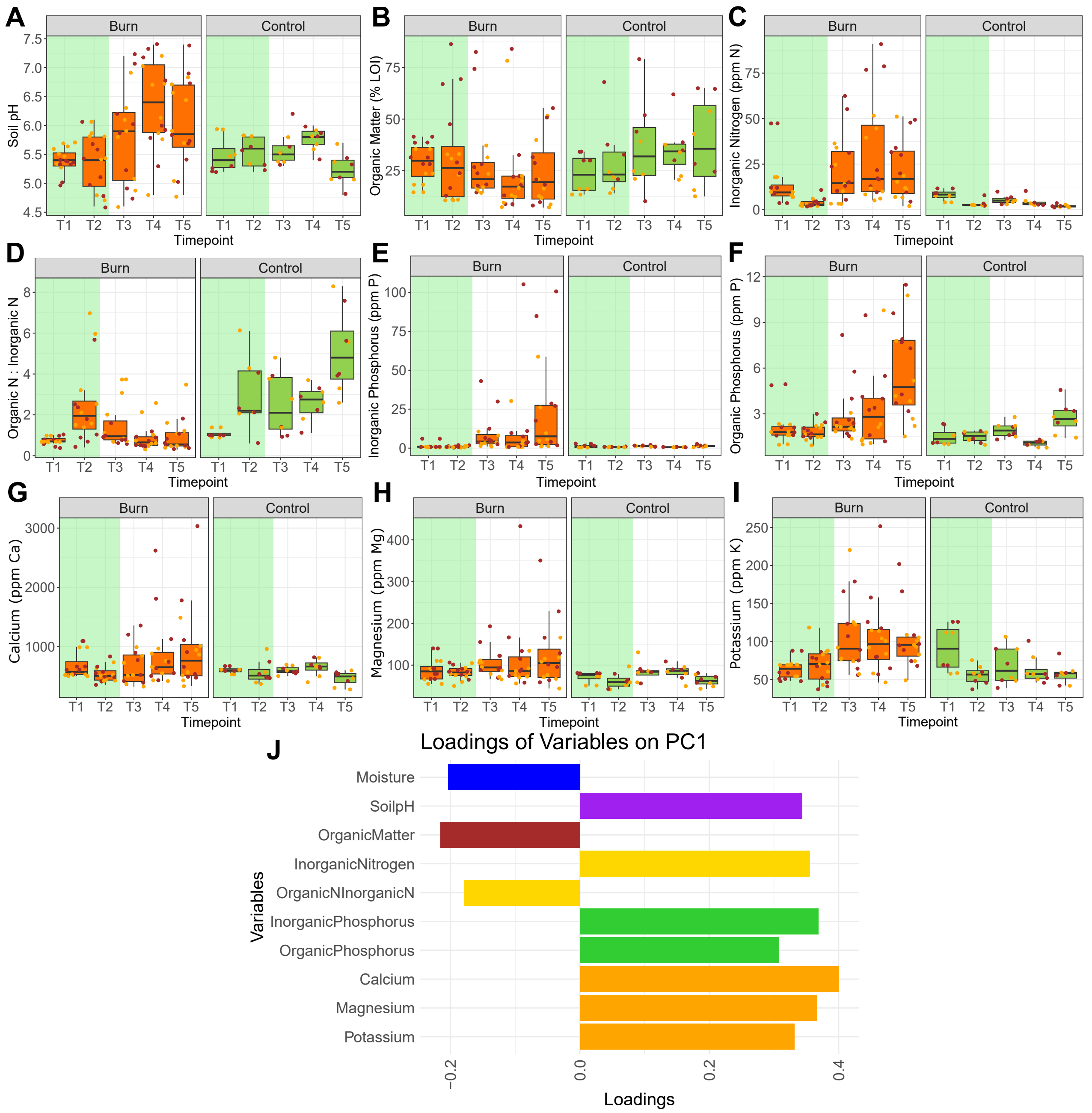
A
B
C
D
E
F
G
H
I
J
Burn
Control
Soil pH
Organic Matter (% LOI)
Inorganic Nitrogen (ppm N)
Organic N : Inorganic N
Inorganic Phosphorus (ppm P)
Organic Phosphorus (ppm P)
Calcium (ppm Ca)
Magnesium (ppm Mg)
Potassium (ppm K)
Timepoint
T1
T2
T3
T4
T5
Loadings of Variables on PC1
Variables
Moisture
SoilpH
OrganicMatter
InorganicNitrogen
OrganicNInorganicN
InorganicPhosphorus
OrganicPhosphorus
Calcium
Magnesium
Potassium
Loadings
−0.2
0.0
0.2
0.4

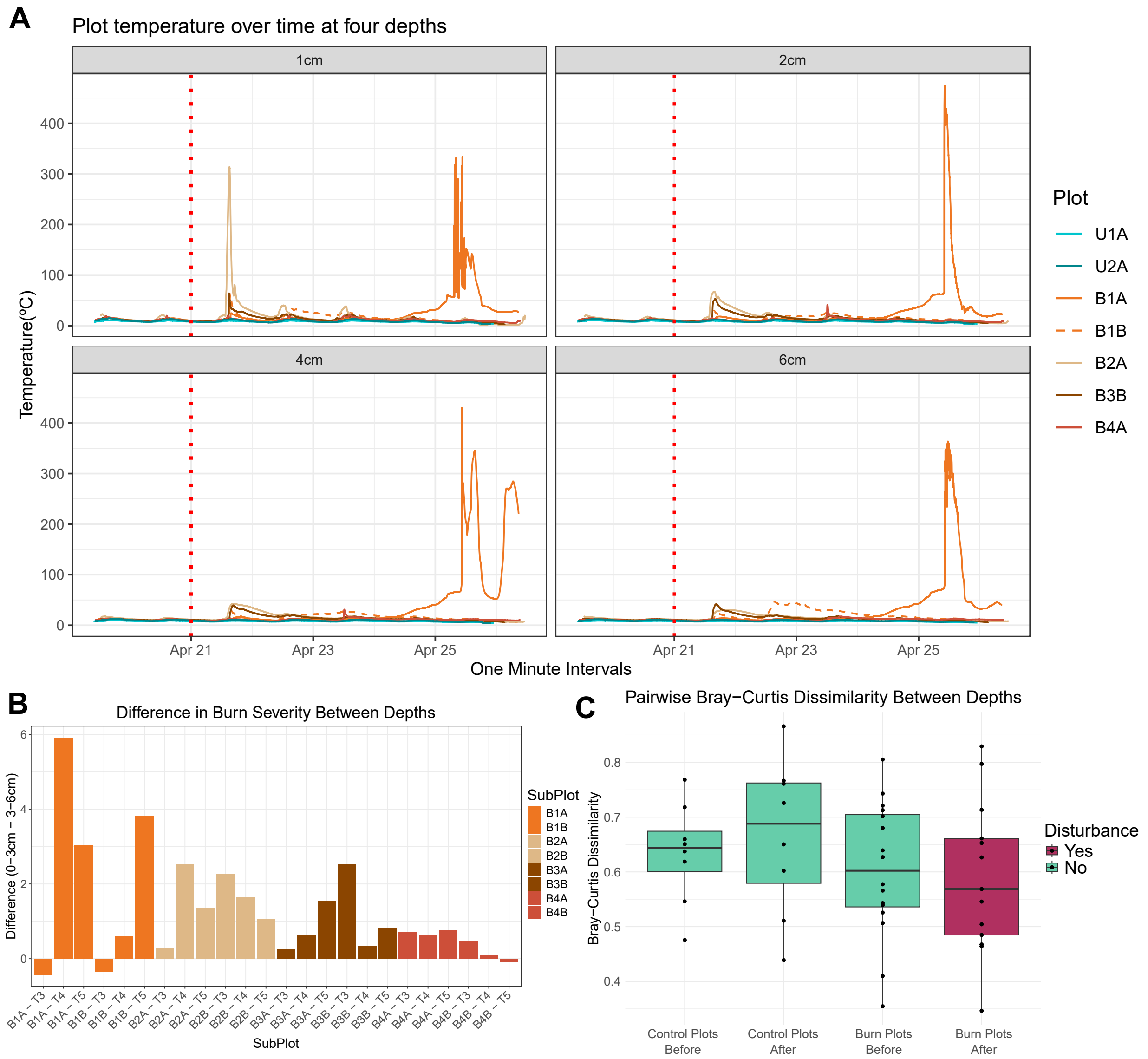

A
Plot temperature over time at four depths
1cm
2cm
4cm
6cm
Temperature(ºC)
One Minute Intervals
Apr 21
Apr 23
Apr 25
Plot
U1A
U2A
B1A
B1B
B2A
B3B
B4A
B
Difference in Burn Severity Between Depths
Difference (0−3cm − 3−6cm)
SubPlot
B1A − T3
B1A − T4
B1A − T5
B1B − T3
B1B − T4
B1B − T5
B2A − T3
B2A − T4
B2A − T5
B2B − T3
B2B − T4
B2B − T5
B3A − T3
B3A − T4
B3A − T5
B3B − T3
B3B − T4
B3B − T5
B4A − T3
B4A − T4
B4A − T5
B4B − T3
B4B − T4
B4B − T5
B1A
B1B
B2A
B2B
B3A
B3B
B4A
B4B
C
Pairwise Bray−Curtis Dissimilarity Between Depths
Bray−Curtis Dissimilarity
Control Plots Before
Control Plots After
Burn Plots Before
Burn Plots After
Disturbance
Yes
No

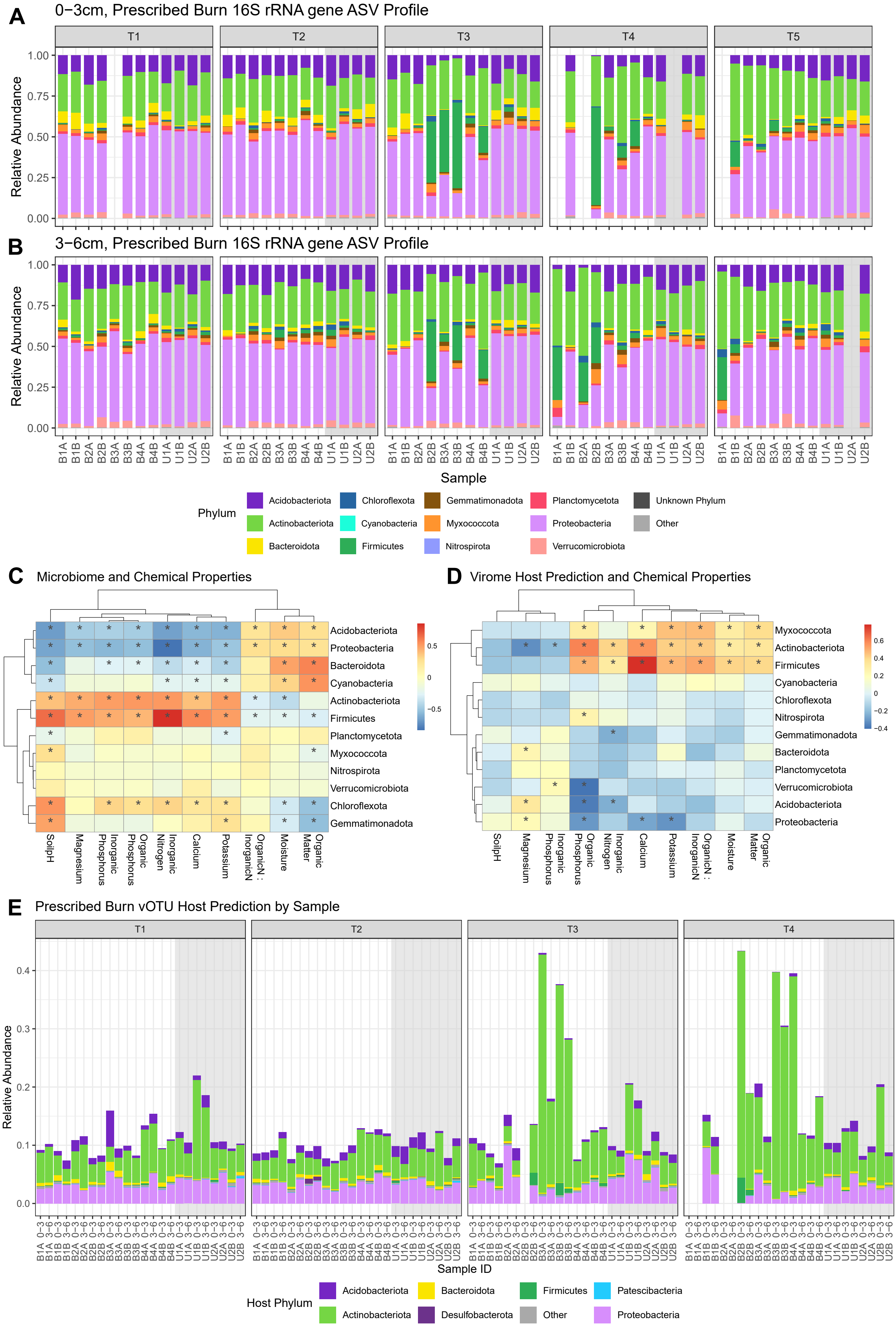
A
0−3cm, Prescribed Burn 16S rRNA gene ASV Profile
T1
T2
T3
T4
T5
Relative Abundance
1.00
0.75
0.50
0.25
0.00
B
3−6cm, Prescribed Burn 16S rRNA gene ASV Profile
B1A B1B B2A B2B B3A B3B B4A B4B U1A U1B U2A U2B
Sample
Phylum
Acidobacteriota
Actinobacteriota
Bacteroidota
Chloroflexota
Cyanobacteria
Firmicutes
Gemmatimonadota
Myxococcota
Nitrospirota
Planctomycetota
Proteobacteria
Verrucomicrobiota
Unknown Phylum
Other
C
Microbiome and Chemical Properties
0.5
0
−0.5
Acidobacteriota
Proteobacteria
Bacteroidota
Cyanobacteria
Actinobacteriota
Firmicutes
Planctomycetota
Myxococcota
Nitrospirota
Verrucomicrobiota
Chloroflexota
Gemmatimonadota
SoilpH
Magnesium
Inorganic Phosphorus
Organic Phosphorus
Inorganic Nitrogen
Calcium
Potassium
OrganicN : InorganicN
Moisture
Organic Matter
D
Virome Host Prediction and Chemical Properties
0.6
0.4
0.2
0
−0.2
−0.4
Myxococcota
Actinobacteriota
Firmicutes
Cyanobacteria
Chloroflexota
Nitrospirota
Gemmatimonadota
Bacteroidota
Planctomycetota
Verrucomicrobiota
Acidobacteriota
Proteobacteria
SoilpH
Magnesium
Inorganic Phosphorus
Organic Phosphorus
Inorganic Nitrogen
Calcium
Potassium
OrganicN : InorganicN
Moisture
Organic Matter
E
Prescribed Burn vOTU Host Prediction by Sample
T1
T2
T3
T4
Relative Abundance
0.4
0.3
0.2
0.1
0.0
B1A 0−3 B1A 3−6 B1B 0−3 B1B 3−6 B2A 0−3 B2A 3−6 B2B 0−3 B2B 3−6 B3A 0−3 B3A 3−6 B3B 0−3 B3B 3−6 B4A 0−3 B4A 3−6 B4B 0−3 B4B 3−6 U1A 0−3 U1A 3−6 U1B 0−3 U1B 3−6 U2A 0−3 U2A 3−6 U2B 0−3 U2B 3−6
Sample ID
Host Phylum
Acidobacteriota
Actinobacteriota
Bacteroidota
Desulfobacterota
Firmicutes
Other
Patescibacteria
Proteobacteria
